# SLN melanoma micrometastasis predictivity of nodal status: a long term retrospective study

**DOI:** 10.1186/1756-9966-32-47

**Published:** 2013-08-01

**Authors:** Emilia Migliano, Barbara Bellei, Flavio Andrea Govoni, Giovanni Paolino, Caterina Catricalà, Stefania Bucher, Pietro Donati

**Affiliations:** 1 Department of Plastic and Reconstructive Surgery, San Gallicano Dermatologic Institute, Rome, Italy; 2 Laboratory of Cutaneous Physiopathology, San Gallicano Dermatologic Institute, Rome, Italy; 3 Department of Maxillofacial Surgery, San Filippo Neri Hospital, Rome, Italy; 4 Dermatopathology Unit, San Gallicano Dermatologic Institute, Rome, Italy; 5 Department of Dermatology-Oncology, San Gallicano Dermatologic Institute, Rome, Italy

**Keywords:** Sentinel lymph node, SLN, CLND, Starz classification, Melanoma micrometastasis, Nodal status

## Abstract

**Background:**

Completion lymph node dissection (CLND) is the gold standard treatment for patients with a positive sentinel lymph node (SLN) biopsy. Considering the morbidity associated with CLND it is important to identify histological features of the primary tumor and/or of SLN metastasis that could help to spare from CLND a subset of patients who have a very low risk of non-SLN metastasis. The objective of this study is to identify patients with a very low risk to develop non-SLNs recurrences and to limit unnecessary CLND.

**Methods:**

A retrospective long-term study of 80 melanoma patients with positive SLN, undergone CLND, was assessed to define the risk of additional metastasis in the regional nodal basin, on the basis of intranodal distribution of metastatic cells, using the micro-morphometric analysis (Starz classification).

**Results:**

This study demonstrates that among the demographic and pathologic features of primary melanoma and of SLN only the Starz classification shows prognostic significance for non-SLN status (p<0.0001). This parameter was also significantly associated with disease-free survival rate (p<0.0013).

**Conclusion:**

The Starz classification can help to identify, among SLN positive patients, those who can have a real benefit from CLND. From the clinical point of view this easy and reliable method could lead to a significant reduction of unnecessary CLND in association with a substantial decrease in morbidity. The study results indicate that most of S1 subgroup patients might be safely spared from completion lymphatic node dissection. Furthermore, our experience demonstrated that Starz classification of SLN is a safe predictive index for patient stratification and treatment planning.

## Introduction

The sentinel lymph node (SLN) is the first lymph node reached by metastasizing cancer cells from a primary tumor. The lymphatic metastasis in melanoma always proceed sequentially involving cancer cell spreading from the primary site to regional nodes then to distant sites. In 1992 Morton et al. have demonstrated that it is rare that melanoma cells skip the sentinel lymph node and metastasize in other nodes [1]. Consequently, since its introduction into clinical practice, SLN biopsy has become a widely accepted procedure for predicting the status of regional lymph nodes [2,3]. The presence of SLN metastases is the strongest prognostic factor for melanoma and the histological status of the sentinel node has repeatedly shown to provide excellent prognostic information with respect to cancer spreading, disease–free and overall survival rate [4]. Current standards of practice suggest completion lymphatic node dissection (CLND) for all the patients with a positive SLN, whereas patients with negative SLN are considered to be at lowest risk of further lymph node extension. CLND aims to increase the local control of disease, survival improvement as well as staging patients. However, several studies have also demonstrated that only 20% of patients with a positive SLN will have further (Non-SLN) metastasis at CLND [5,6]. Although the impact of early dissection of subclinical micrometastatic nodes is well documented on the overall survival rate [7-9], most of the patients don’t present nodal involvement. Moreover, considering the morbidity associated with CLND (paresthesias, wound infection, seroma and lymphoedema) it will be important to identify histological features of the primary tumour and/or of SLN metastases that could help to spare from CLND a subset of patients who are unlike to have metastatic non-SLNs and thus will not take any benefit from further nodal dissection.

The Starz classification is a micromorphometric analysis of the SLNs based on two parameters: the number of SLN slices, that contained melanoma cells, and the maximum depth of cellular invasion, measured as the maximum distance in millimetres between intra-nodal tumour cells and the inner margin of SLN capsule [8].

Our study was designed to define the risk of additional metastasis in the regional nodal basin on the basis of SLN micro-morphometric study, in order to identify patients with the lowest risk of tumour metastasis in NSLNs. Moreover, we retrospectively evaluated the disease-free survival (DFS) rate and the overall survival (OS) rate of patients, considering several clinical and pathological aspects of primary melanoma compared with the findings of micro-morphometric analysis performed on the excised lymphatic nodes.

## Methods

### Patients

Between 2000 and 2005, 537 consecutive patients with primary cutaneous melanoma that underwent to SLN biopsies were identified from a prospectively maintained departmental database comprising 685 patients. Among these, 100 SLN positive patients (18.6%) subsequently undergone to CLND were initially enrolled for this study. However, the availability of the original specimens for histopathologic re-examination and a full documented post-operative period (at least five years) restricted the patient group to 80 subjects. All data from patients undergone sentinel lymph node biopsy, regardless of gender, age and localizations were retrieved from the pathology database of Dept. of Plastic Surgery and of the Dept. of Dermatopathology of the “Dermatological Institute San Gallicano” of Rome, comprising more than 900 patients from a 13-years period (1997–2010). To obtain a full post-operative period of at least five years we selected 80 subjects showing positive SLN treated between 2000 and 2005. Most patients were followed in the Departments of Plastic Surgery and the data concerning their evolution were available in their medical records. For those who interrupted their follow-up, the physician in charge of follow-up was interviewed systematically to get the latest status. Survival was calculated from the date of the initial excision of the primary tumor.

### SLN procedure

All patients underwent preoperative lymphoscintigraphy to ascertain the number and location of regional nodal basins at risk for metastatic disease. The lymphoscintigraphy was performed the day before or the same day of surgery by intradermal injection of technetium-99-labeled nanocolloid. Under a general anaesthesia or neuroleptanalgesia, blue patent V (0.5-1 ml) was injected intradermally around the excisional scar. Sentinel lymph nodes were identified intraoperatively by their blue colour and radioactivity detected with hand-held gamma probe. All blue nodes and all radioactive nodes (hottest) were considered sentinel and were removed. All patients presenting a positive SLN underwent within four weeks to a CLND.

### Histopathological examination

SLNs were fixed in 4.5% formaldehyde for 24 hours. Then three-dimensional measurement and macroscopic characteristics were evaluated for every lymph node. Lymph nodes were cut parallel to the longest axis into slices about 1 mm thickness and embedded in paraffin blocks. Four sections (3 μm thick) of each slice were produced with a microtome: the first one was stained with haematoxylin-eosin, and the subsequent for the immuno-hystochemistry with S100, HMB45 and MART1 antibodies [9,10].

### Starz staging

According to the Starz classification [8,11,12] all patients were divided into three categories based on the number of positive sections (*n*) and the maximum distance from the interior margin of the biggest metastatic group to the capsule of the SN (*d*) as follows: S1 for peripheral involvement (1<*n*<2 and *d* <*0*.*3 mm*), S2 for extended or multifocal involvement (*n*>*2* and 0.3<*d*<*1 mm*) and S3 classifying metastatic invasion deeper than 1 mm below the capsular level (*d*>*1 mm*) [8,11,12].

### Statistical analysis

An independent biostatistician performed statistical evaluation. Patient’s characteristics included: demographic data (age and sex) and histological features of the primary melanoma (Breslow thickness, Clark level, ulceration and histological subtype); while for the sentinel lymph node included the number of sentinel lymph node removed, the pattern of invasion and the invasion depth of metastatic cells in the sentinel lymph node (Starz Classification). For statistical analysis parametric tests were applied: Hazard Ratio and 95% Confidence Interval were used to study the test and overall survival rate. Kaplan-Meier curves were used to estimate significance in OS differences. Significance for all statistical tests was defined as p values <0.005.

## Results

**Figure 1:**
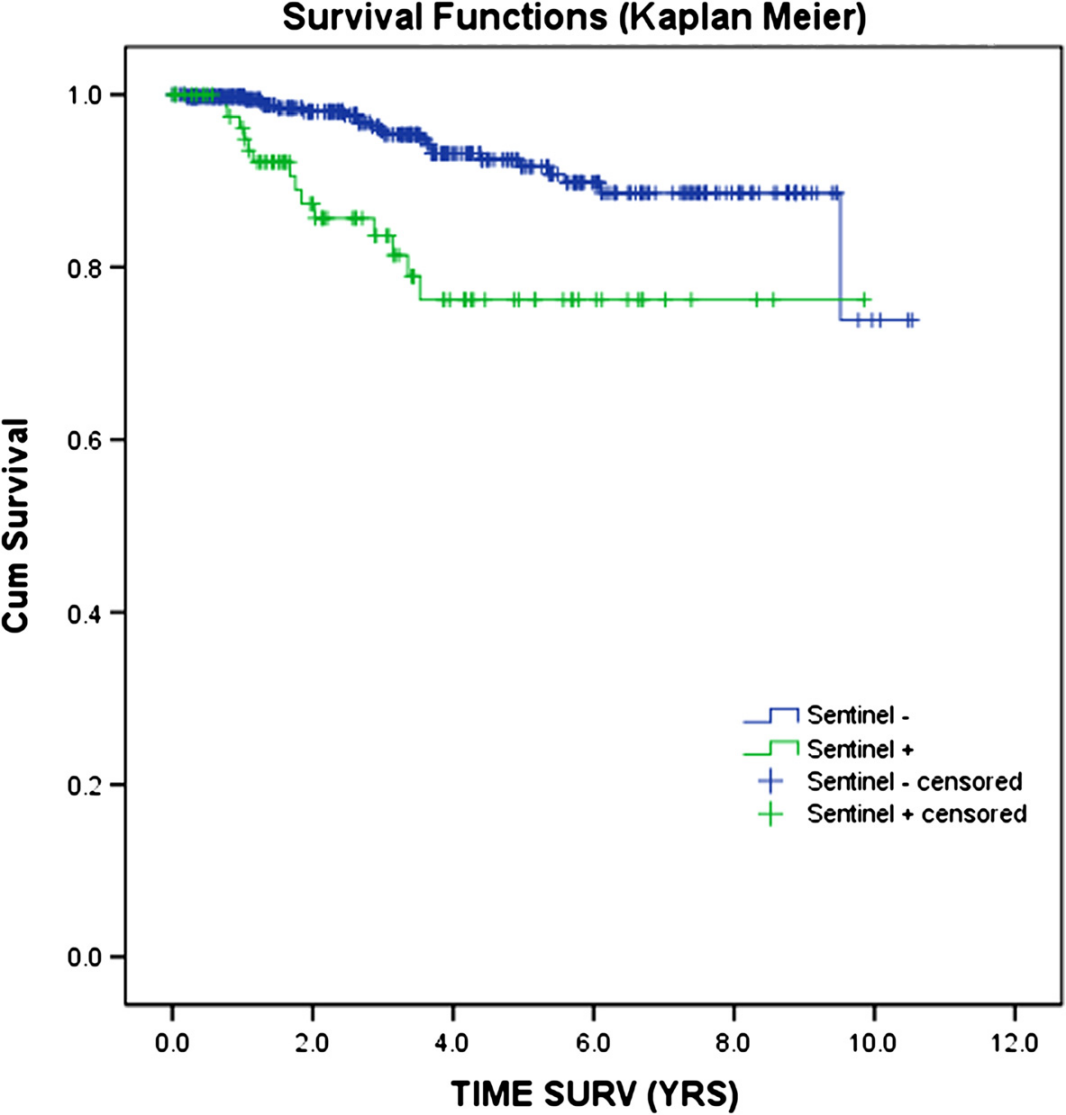
**Kaplan-Meier survival curve for patients undergoing successful CLND.** The ten-years overall survival (OS) showed a significant shorten survival in SLN-positive patients than in SLN-negative patients (p<0.0001). Mean survival time (8.01±0.44 yrs for SLN+ and 9.61±0.21 yrs for SLN-).

In this study we have enrolled 80 patients, 46 (57%) were males and 34 (43%) were females (mean age 48 years; range of 20–83 years). The mean Breslow thickness of the primary melanoma was of 3.0 mm (range 0.4-6.0 mm); 3 patients (4%) were of Clark II, 21 (26%) were of Clark III, 52 (65%) were of Clark IV and 4 (5%) of Clark V. Melanoma subtype included nodular (36%), superficial spreading (47%), and polypoid (17%). More than half of the tumors were ulcerated (51%). Regarding the regional distribution of SLN biopsies 36 were axillary (45%), 32 groin (40%), 8 (10%) present a double basin (7axillary+groin and 1 axillary+supraclavear), and 4 of the neck (5%). CLND found at least one positive non-SLN in 15 cases (19%). The median follow-up was 78 months (range 60–120 months). During the follow-up period only 5 patients (6%) had a loco-regional recurrence. From the 80 enrolled cases, 69 (86%) were alive without evidence of disease at the time of this writing. According with the literature, Kaplan Meier curve, used to evaluate overall survival (OS), showed a significant shorten survival in SLN-positive patients than in SLN-negative patients (p<0.0001; chi-square test) (Figure 1). To better analyze the data, patients were divided according to the number of lymph nodes excised after finding micro-morphometric metastasis in SLN. In particular, in 9 patients (11%) were excised only one lymph node, in 24 patients (30%) were excised two lymph nodes, in 38 patients (48%) were excised three lymph nodes while in 9 (11%) were excised more than 3 lymph nodes (Table 1). Patients were also divided further by the number of positive NSLNs: 47 patients (59%) presented one positive lymph node, 15 patients (19%) two positive lymph nodes, 12 patients (15%) presented 3 positive lymph nodes whereas for 6 patients (7%) the positive lymph nodes were more than 3 (Table 2).

**Table 1:** Results for number of excised SLN

**EXCISED SLN (N)**	**N Patients**	**%**
1	9	11%
2	24	30%
3	38	48%
>3	9	11%

**Table 2:** Results for number of positive SLN

**DISEASE-POSITIVE SLN (N)**	**N Patients**	**%**
1	47	59%
2	15	19%
3	12	15%
>3	6	7%

Regarding the Starz classification we found that 40 patients (50%) were classified as S1, 15 (19%) as S2 and 25 (31%) as S3 (Table 3). In patients without NSLNs involvement, 40 SLNs (61%) were classified as S1, 9 (14%) as S2, while 16 SLNs (25%) were classified as S3. On the other hand, in NSLNs with metastasis, we reported 9 SLNs (60%) were classified as S3 and 6 SLNs (40%) were classified as S2. None of the 40 patients of the S1 group presented NSLN metastasis. The occurrence of at least one melanoma-positive non-SLN significantly increased from 0 (of 40 in S1 SLNs) to 6 (of 15 in S2 SLNs) up to 9 (of 25 in S3 SLNs) (p=0.0124; chi-square test). Moreover, it is important to highlight that among the parameters studied the univariate analysis indicated a significant association of NSLNs metastasis only with the Starz classification (p<0.0001; chi-square test) (Table 4). The mean Breslow thickness was 2.6 mm for S1 group, 2.8 mm for the S2 group, and 3.9 mm for the S3 group. The highest percentage of ulcerated primary tumor was found in the S3 patients group (S1 56%, S2 40%, S3 83%). Concerning the distribution of melanoma subtypes we found: in the S1 group 24 of 40 (60%) were SSM, 11 of 40 (27.5%) nodular and 5 of 40 (12.5%) polypoid; in the S2 group 8 of 15 (54%) were SSM, 5 of 15 (33%) nodular, 2 of 15 (13%) polypoid; in the S3 group 4 of 25 (16%) were SSM, 14 of 25 (56%) nodular and 7 of 25 (28%) polypoid. Distant metastasis were present in 2 patients S1 (5%), in 2 patients S2 (13%) and in 2 patients with S3 (8%). S-classification results are summarized in Table 5. The univariate analysis of disease-free survival (DFS) showed no significant association with the variables considered in the study (sex, age, Breslow thickness, and number of positive SLNs) with the exception of S-classification (OR 7.4 [95% CI 1.94, 28.24]; p=0.013; chi-square test) (Table 6). The overall OS rate was 86%, among the 11 patients dead we observed the following distribution: in the S1 group 3 of 40 patients (7,5%), in the S2 group 2 of 15 patients (13%), and in the S3 group 6 of 25 patients (24%). The OS analysis showed significant association only with the Breslow thickness (OR 3.08 [95% CI 0.75, 12.61]; p=0.002) (Table 7).

**Table 3:** **Results of S**-**classification for patients in this study**

**S-classification**	**N Patients**	**%**
S1	40	50%
S2	15	19%
S3	25	31%

**Table 4:** **Univariate analysis of sex**, **age**, **Breslow thickness**, **number of positive lymph nodes and S**‒**classification**

	**Disease-negative CLND (n=15)**		**Disease-positive CLND (n=15)**		**univivariate analysis**
	**No**	**%**	**No**	**%**	**P value**
**SEX**					
male	39	60%	7	47%	0.346
female	26	40%	8	53%	
**AGE**					
Mean ±SD	48.5±16.3		47.9±11.9		0.880
Range	20–83		30–67		
**BRESLOW THICKNESS**					
Mean ±SD	2.8±1.2		2.7±1.4		0.744
Range	1.0–6.0		0.4–4.1		
**N of positive SLN**					
1	46	71%	13	87%	0.207
>1	19	29%	2	13%	
**STARZ CLASSIFICATION**					
S1	40	61%	0	0%	**0**.**0001**
S2	9	14%	9	40%	
S3	16	25%	6	60%	

**Table 5:** **Tumour characteristics of 80 patients with cutaneous melanoma who underwent CLND divided according to the S**-**classification**

**Histologic type**								
**S-group**	**Ulceration %**	**Breslow (mm)**	**SSM %**	**Nodular %**	**Polipoid %**	**CNLD + %**	**Distal Mestastasis %**	**Death**
**S1**	56%	2.6	60%	27.5%	12.5%	0%	5%	7.5%
**S2**	40%	2.8	54%	33%	13%	40%	13%	13%
**S3**	83%	3.9	16%	56%	28%	36%	8%	24%

**Table 6:** Disease free survival analysis

**DISEASE-FREE SURVIVAL RATE**			
	**HR**	**95% C.I.**	**P value**
**SEX**			
Male	1		
Female	3.28	0.366-29.455	0.288
Age(Y)*	1.004	0.950-1.062	0.874
Breslow (mm)*	3.16	0.678-11.517	0.081
**No positive SLN**			
1	1		
>1	1.672	0.279-10.006	0.54
**STARZ CLASSIFICATION**			
S1	1		
S2-S3	7.4	1.938-28.244	**0.0013**

*C.I.* confidential interval, *HR* Harzard ratio, *as continuous variable.

**Table 7:** Overall survival analysis

**OVERALL SURVIVAL RATE**			
	**HR**	**95% C.I.**	**Pvalue**
**SEX**			
Male	1		
Female	1.692	0.588–4.867	0.33
Age(Y)*	1.02	0.986–1.055	0.244
Breslow(mm)*	7.42	2.031–27.119	**0.002**
**No Positive SLN**			
1	1		
>1	1.727	0.576–5.179	0.33
**STARZ CLASSIFICATION**			
S1	1		
S2-S3	3.083	0.753–12.613	0.104

*C.I.* confidential interval, *HR* Harzard ratio, *as continuous variable.

## Discussion

Negative SLN biopsy findings are well known prognostic factors. Other ways, the positivity to a SLN biopsy lead the patient to a completion lymph node dissection (CLND) and approximately the 35%–50% of SLN positive patients die within 5 years [13-15]. Morton et al. demonstrated a highly significant survival benefit in SLN-positive patients who had received CLND (5-year survival rate 72%) compared to SLN-positive patients who had received delayed ELND (Elective LND) after clinical detection of lymph node metastasis (5-year survival rate 52%) [16].

However, at the moment, there is no consensus on the benefit of a completion dissection in melanoma patients. As reported in literature, only the 14%-18% of positive patients will harbour further disease in the affected basin [14-17]. Only patients with secondary involvement in NSLNs find benefit in a CLND while a large percentage of patients (NSLNs negative) will increase only the morbidity rate due to this surgical procedure [18]. In this respect it will be of primary importance to identify histological biomarkers (relative to patient, tumour, and SNL characteristics) that can safely predict an additional risk of NSLN recurrence in SLN positive patients. In this way we will be able to increase the disease-free survival and the overall survival rate lowering at the same time the morbidity rate. In our opinion the key point would be to recommend CLND only to those patients who have an high predictive risk of NSLN positivity, using a patient selection criteria as currently stated in the treatment of breast cancer, where patients with sub-micrometastasis (< 0.2 mm) in the SLN are spared from axillary CLND, due to the very low risk of nodal recurrence [19-22]. In melanoma the Breslow thickness and the ulceration of the primary tumour, the number of positive SLNs and tumour penetrative depth inside the SLN are significant prognostic factors of high risk NSLNs positivity [14,15,22-26]. However, statistical data reviewed from the literature on these factors are still very poor so that currently none of these parameters can give a safe a reliable prognostic indication on NSLNs status. Previous studies have shown that several characteristics of deposits of metastatic melanoma in SLNs correlate with the presence of tumour in NSLNs in subsequent CLND specimens [17,21-24]. In our study, the microanatomic features of the SLNs metastasis, particularly the tumour penetrative depth of the deposit (according with Starz classification) and several clinic-pathologic data were analyzed looking for a predictive marker for NSLN involvement. Among 80 cases underwent CLND, 15 patients (19%) had NSLN positivity, while the remaining 65 (81%) had no metastases, according to the data reviewed from the literature [13,14,18,27-30]. Patients presenting a positive CLND were all classified as S2 or S3 at the SLN histological micro-morphometric analysis confirming that Starz classification is an indicative factor of high risk of regional nodal recurrence. (Table. 6; *p* value= 0,0013.) The evaluation of “median primary tumour thickness” factor resulted, in our study, not statistically significant (*p* value=0.7436) on NSLNs metastasis, but well correlated to the OS (overall survival rate - Table 7; *p* value=0,02). The predictive value of “tumour ulceration” factor on NSLN involvement has been found in some previous studies, but not confirmed by others, thus indicating a great deal of variability which limits the drawing of definite conclusions [31-38]. In our study, the ulceration of primary lesion was present in 41 patients (51%). Evaluating ulceration factor in S-subgroups 56% of S1, 40% of S2 and 83% of S3 patients had ulcerated lesions. Among the 11 patients who died for melanoma metastasis the ulceration factor was present in 9 (81%). It is interesting to note that inside the group of died patients 6 (55%) were classified as S3, 2 (18%) as S2 and 3 (27%) as S1. The analysis of S1 dead patients revealed that everyone presented peculiar characteristics: one patient had two different SLN compromised, another patient presented severe ulceration of the primary lesion, while the third patient had an high Breslow thickness, nodular type, primary melanoma. These results outline the relevance of clinical biomarkers that can be useful, in correlation to the histological markers, to predict S1 patients clinical outcome. It should be reported, that Reeves et al. [26] proposed the ratio size of metastases on SLN/ulceration (S/U score) as predictor factor of NSLNs status, while Frankel et al. [27] utilized the relation between the thickness of primary tumour and the surface area, measured in percentage, of the metastases on SLN.

According with previous studies [2,14,16,17,27] and the recent study of Nagaraja [38], where it is shown a very accurate and extensive meta-analysis involving several predictive factors to determine the risk of lymph node metastasis, our data confirmed that about 20% of SLN positive patients undergone CLND present an additional lymphatic involvement. At the moment, according to the staging guidelines of the American Joint Committee on Cancer (AJCC) the most important prognostic factor in patients affected by melanoma is the SLN status [28-31]. The current standard treatment for SLN positive patients is the completion lymphatic node dissection. Within the last few years, several studies have been conducted to determine whether some patients could be classified as low risk of further nodal metastasis according to the type of involvement of the SLN. Furthermore, the overall data published [11,16,21,29] and the present study evidenced that the prognosis of patients is determined not only by the presence of melanoma cells in SLNs but also by a micro-morphometric characterization of SLNs according to the Starz classification. On these bases some Authors suggested the possibility to avoid the CLND to a subgroup of selected patients [30-34]. Already in few centres, patients with SLN tumour deposits <0.1mm in maximal dimension can choose if undergo CLND or clinical nodal follow-up [16,18,33-38]. In our report, using univariate analysis, we confirmed the prognostic relevance of Starz classification suggesting that patients classified as S1 could safely spare to the CLND. None of S1 patients presented CLND positivity, suggesting that the increased morbidity associated with complete nodal dissection could be avoided in this group of patients. Moreover, the DFS of S1 patients was significantly higher than S2-S3 patients (p value 0.0013).

In conclusion, our results showed that, among the 80 SLN positive melanoma patients studied, 65 (81%) underwent to CLND in absence of an evident benefit but increasing only the morbidity. NSLNs metastases were found only in 15 patients (19%). None of the S1 patient had positive NSLN. Considering that we recorded three dead patients among the S1 subgroup in absence of NSLN involvement, our future study will aim to select important biomarkers that, in combination with S-classification, could help to select a S1 subgroup presenting major risk of disease progression. Interestingly, while this research was in progress, Veenstra et al., reported a positive 5-years experience for 16 melanoma patients classified as Starz level 1 that did not undergo completion lymphatic node dissection [33]. Although further investigations are needed, on larger and multicentric studies, we think that our observations can contribute to suggest the way to find a clinically reliable technique (i.e. an algorithm of the mentioned factors), and an easy application method to identify, among melanoma patients, those who present the higher risk of NSLN recurrence [37,38]. In our opinion this selection will provide a more accurate depiction of prognosis and will help to define subsequent recommendations for the treatment and the follow-up care.

## Competing interest

The authors declare that they have no competing interest.

## Authors’ contributions

EM was the research leader, conceived the study, collected the clinical informations, drafted and revised the manuscript. BB and GP participated in clinical data collection-analysis and in manuscript drafting. FAG performed the critical revision of the research data and participated in the writing of the final manuscript. CC and SB contributed to the financial support of the research and were involved in the final approval of the manuscript. PD performed the revision of all histopathological and immuno-hystochemical examinations included in this research. All the authors read and approved the manuscript.
